# The Health Sector Evolution Plan and the Technical Efficiency of Public Hospitals in Iran

**Published:** 2019-09

**Authors:** Edris KAKEMAM, Hossein DARGAHI

**Affiliations:** 1. Iranian Center of Excellence in Health Management, School of Management and Medical Informatics, Tabriz University of Medical Sciences, Tabriz, Iran; 2. Department of Management Sciences and Health Economics, School of Public Health, Tehran University of Medical Sciences, Tehran, Iran; 3. Health Information Management Research Center, Tehran University of Medical Sciences, Tehran, Iran

**Keywords:** Data envelopment analysis, Efficiency, Health care reform, Public hospital, Iran

## Abstract

**Background:**

Iranian public hospitals have been excessively changing during the healthcare reform since 2014. This study aimed to examine the technical efficiency of public hospitals during before and after the implementation of Health Sector Evolution Plan (HSEP) and to determine whether, and how, efficiency is affected by various factors.

**Methods:**

Forty-two public hospitals were selected in Tehran, Iran, from 2012 to 2016. Data envelopment analysis was employed to estimate the technical and scale efficiency sample hospitals. Tobit regression was used to relate the technical efficiency scores to seven explanatory variables in 2016, the last year.

**Results:**

Overall, 24 (57.1%), 26 (61.9%), 26 (61.9%), 24 (57.1%) and 21 (50%) of the 42 sample hospitals ran inefficiently in 2012 to 2016, with average technical efficiency of 0.859, 0.836, 0.845, 0.905 and 0.934, respectively. The average pure technical efficiency in sample hospitals increased from 0.860 in 2010 (before the HSEP) to 0.944 in 2012 (after the HSEP). Tobit regression showed that average length of stay had a negative impact on technical efficiency of hospitals. In addition, bed occupancy rate, ratio of beds to nurses and ratio of nurses to physicians assumed a positive sign with technical efficiency.

**Conclusion:**

Despite government support, public hospitals operated relatively inefficien. Managers can enhance technical efficiency by increasing bed occupancy rate through shortening the average length of stay, proportioning the number of doctors, nurses, and beds along with service quality assurance.

## Introduction

Iran is a middle-income country with a population of more than 81 million people. According to WHO statistics, share of total expenditure of gross domestic product (GDP) in Iran increased from 4.6 in 2000 to 6.9 in 2014 (1). The healthcare system in Iran consists of three levels: primary health care, secondary and tertiary. At primary care level, rural areas have health houses and rural health centers and basic healthcare services provided by local health workers called Behvarzes. In the urban areas, secondary and tertiary healthcare is delivered in hospitals. The share of public sector of 924 Iranian‘s Hospitals is 570 hospitals (70.0% of total hospital beds) (2, 3). Therefore, the performance of public hospitals has too much effect on the wellbeing of the Iranian people.

Hospitals play a significant role in the Iranian healthcare system and they are the main consumers of resources. More than 50% of health care system costs are allocated to hospitals (4, 5). However, increase in spending on health care has not been proportionate to the increase in public access. Moreover, Iranian health system suffers from inadequate response to increased demands and there is a health gap between urban and rural areas (6).

In Iran, more than 50% of total healthcare expenditures are financed via out of pocket payments (OOP) (7) and there were some challenges associated with productivity and efficiency of public hospitals (8). In addition, public hospitals are faced with problems such as low bed occupancy rate (9), informal payments (10, 11) and poor quality of care (8, 12).

Therefore, Iranian Ministry of Health and Medical Education (MOHME) launched a series of reforms called the Health Sector Evolution Plan (HSEP) in 2014. The HSEP is a stepwise national plan which focuses on three approaches of financial protection of people, provision of access to health services, and promotion of the quality of services (13–15).

The first phase of the HSEP focused on hospitals affiliated to MOHME, the second phase of HSEP which started on May 22, 2014, was conducted with focus on primary health care and public health areas. The third phase of the HSEP began on Sep 29, 2014, which updated tariffs of health services in all parts of the health system (15).

The first phase has six main area of action, including providing free basic health insurance to all uninsured individuals by Health Insurance Organization (HIO) through public funding, reducing OOP payments in public hospitals, financial protection of patients whit specific diseases, provide conditions to encourage medical doctors to stay in deprived areas, improving quality of services delivered in hospitals affiliated to MOHME (through increasing specialists, attaching polyclinics to hospitals to provide better outpatient services and improving hospitals amenities and hoteling services), and compensation to offset the economic burden of the second phase of the targeted subsidies’ law at hospitals affiliated to MOHME (9, 13, 14). In addition, at the first phase of HSEP implementation, some measures were planned and done in line with promotion of natural vaginal delivery and infertility treatments and improving the emergency medical services.

The HSEP financed through increased annual budget health sector (approximately 59% in 2015 in comparison with 2014), resources of the targeted subsidies’ law (10% of total subsidies) and 1% of value-added tax (9, 13, 14).

These reforms have had substantial positive results. For instance, HSEP implementation has reduced the OOP payments for inpatient services and eradicated informal payments to physician (15). Furthermore, people’s access to and utilization of hospital services increased (13). HIO coverage has been expanded to cover more than 95% of people 2014 to 2016; health houses, rural health centers, and public hospitals have been strengthened. For example, 39.000 beds have been rebuilt or upgraded and 21.000 beds in public hospitals were added (21%).

The expansion of HIO coverage and the strengthening of public hospitals lead to a rapid increase in people’s health demands. Despite the positive implications, there are concerns about the stability of the HSEP and its unwanted negative consequences. In this area can be pointed to concerns regarding the economic burden of the program on the public budget and sustainability of the program in the following years, increases tariffs, not achieving the goal of improving service quality due to increased number of unnecessary referrals, and increases in patient’s illegal payments (13).

Assessment of the technical efficiency of public hospitals and factors affecting efficiency are important in these conditions. Assessment of the efficiency using data envelopment analysis (DEA) is a well-known method and is widely used in healthcare (4, 5, 16–18). Therefore, the aim of our study was to examine the technical efficiency of public hospitals during 2012 to 2016, comparing their efficiency before and after the HSEP implementation and finally, to determine the various factors that affect on the technical efficiency.

## Materials and Methods

This descriptive analytic and retrospective study was carried using the data 2012 to 2016 among 42 public hospitals in Tehran, Iran.

### Efficiency evaluation methods

To measure the efficiency, two frontier methodologies, stochastic frontier analysis (SFA) and DEA, have been widely (4, 5, 16, 17). In contrast SFA, DEA method does not require constructing an efficient frontier function, and the information of input prices (17). Therefore, because of convenience and multiple inputs and outputs, we used DEA. As DEA models, Charnes, Cooper, and Rhodes (CRR) and Banker, Charnes, Cooper (BCC) are widely used to assess efficiency. In CCR model (19), production is assumed constant return to scale (CRS) i.e. the increase in inputs leads to a proportional increase in output. When a hospital is active in CRS, technical efficiency is equal to scale efficiency. Bunker (20) presented BCC model. This model assumes that production returns variable to scale (VRS). VRS consists of two dimensions: increasing returns to scale, i.e. 1% increase in inputs will bring about more than 1% increase in outputs, and decreasing returns to scale, i.e. 1% increase in inputs will result in an increase of less than one percent in the output. In this study, an input-oriented DEA model was performed for several reasons. In comparison with output-oriented DEA, in input-oriented DEA models, hospital managers have more control over their inputs than they do over outputs. In addition, the input-oriented model focuses on the minimization of inputs with given outputs that conformed to the character of public hospitals in Iran which their initial goal is not incom generation. Previous empirical research used input orientation model for hospital efficiency evaluation (4, 5, 16, 17).

### Sampling and Data

Data were obtained from the statistical centers of Universities of Medical Sciences in Tehran. All Tehran public hospitals were selected as the research setting and then hospitals without data for the five consecutive years (the time period was chosen for the study) or their data on the selected items were incomplete, were excluded. Finally, 42 public hospitals were selected in the data set. In order to assessment of technical efficiency, the selection of input and output variable were with regards to previous studies (4, 5, 16, 17) and the availability of data. Given the importance of labor and capital in the provision of health services, in our study, the labor variables focused on the number of doctors, nurses, and other staff, and the number of hospital beds were chosen as input variables. Based on previous evidence, we were showed hospital outputs in the study by the number of outpatients, emergency department visits, and the number of inpatient days (16, 17).

### Data Analyses

The descriptive analysis of the inputs and outputs variables was performed using SPSS ver. 23 (Chicago, IL, USA). Technical efficiency was estimated using DEAP ver. 2.1 software. In this study, Tobit regression was used to relate the technical efficiency scores to seven explanatory variables in 2016. Some of the factors that influenced on hospital efficiency were hospital size, teaching status, Bed Occupancy rate (BOR), Average Length of Stay (ALoS), Ratio of Nurses to Physicians (RONTP) and Ratio of Beds to Nurses (ROBTN) (4, 5, 16, 17). Therefore, the estimated empirical model as follows:

$$\begin{array}{l} \text{TE}=\beta 0+\beta 1\text{SES}+\beta \text{2BED}\;\text{group}\;+\beta 3\text{ALoS}+\beta 4\;\text{BOR}+\\ \beta \text{5RONTP}+\beta \text{6ROBTN}+\beta \text{7}\;\text{teaching}\;\text{status}+\mathrm{ɛi} \end{array}$$

Where: TE is the technical efficiency score; Bed is the hospital beds dummy variable is the) size 1=hospitals less than 150 beds; size 2=hospitals with 150–250 beds; size 3= hospitals with more than 250 beds); teaching status dichotomous dummy variable (0=if hospital is teaching; 1=if hospital is non-teaching) and εi is the error term. Tobit regression was estimated using STATA ver. 13 (21). The research protocol was approved by the Review Committee of the School of Public Health of the Tehran University of Medical Sciences. (IR.TUMS.REC 1394.1891)

## Results

**Fig. 1:**
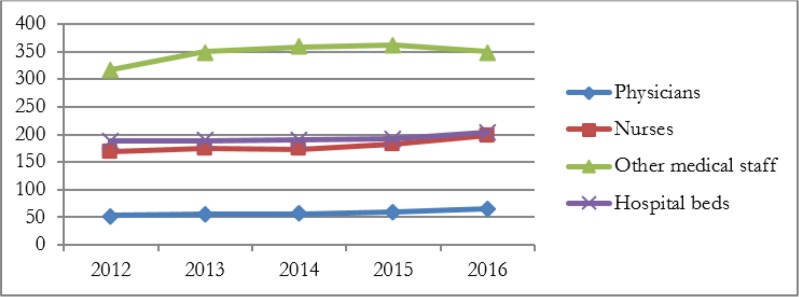
The trend of the number of physicians, nurses, other personnel, and beds from 2012 to 2016

**Fig. 2:**
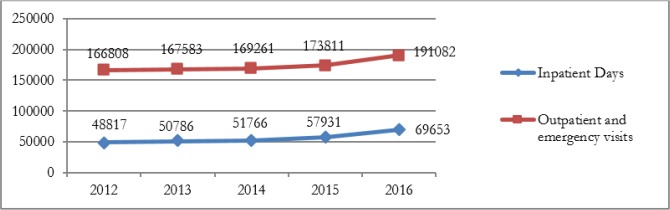
Variations of the number of inpatients d and outpatient and emergency visits from 2012 to 2016

Figures 1 and 2 show changes in inputs and outputs during the study period. The number of inputs (physicians, nurses, and beds) had steady and slightly increase but after the implementation of HSEP, these changes has increased. In contrast, there were some fluctuations in the number of other medical staff during the years 2012 to 2016, such that after the implementation of HSEP has diminished (Fig. 1). The number of inpatient days and outpatient and emergency visits increased from 2014 (after the implementation of HSEP). As shown in Fig. 2, for example, the number of inpatient days increased by 20836, from 48817 to more than 69653. The descriptive statistics of explanatory variables are shown in Table 1.

**Table 1:** Descriptive statistics of explanatory variables during 2012–2016

***Variable***	***2012***	***2013***	***2014***	***2015***	***2016***
Average Length of Stay (Day)					
Mean	7.16	7.37	7.11	7	6.8
Maximum	13.4	14.2	12.7	12.8	11.3
Minimum	4.5	5.2	4.8	4.2	4.1
SD	1.6	1.7	1.5	1.6	1.5
Bed Occupancy Rate (%)					
Mean	73.4	73.9	75.42	76.75	79
Maximum	91.7	90.60	91	92.24	93.1
Minimum	4.50	50.10	53	55.2	55.7
SD	10.9	10.11	9.81	8.73	9.5
Ratio Of Nurses To Physicians					
Mean	3.54	3.44	3.31	3.34	3
Maximum	8.5	8.37	6.44	6.54	5.62
Minimum	1.19	1.11	0.93	0.83	0.77
SD	1.64	1.48	1.27	1.31	1.15
Ratio Of Beds To Nurses					
Mean	1.15	1.12	1.13	1.12	1.07
Maximum	1.88	1.81	1.86	2.12	1.80
Minimum	0.49	0.50	0.57	0.63	0.71
SD	0.36	0.34	0.33	0.33	0.29

The results of the DEA estimation (TE and SE of sample public hospitals) during 2012–2016 are shown in Table 2.

**Table 2:** Technical and scale efficiency of hospitals, and frequency distribution during before and after the implementation of HSEP

	***Before HSEP***						***Implementation of HSEP***			***After HSEP***					
	**2012**			**2013**			**2014**			**2015**			**2016**		
	***TE_CRS_***	***TE_VRS_***	***SE***	***TE_CRS_***	***TE_VRS_***	***SE***	***TE_CRS_***	***TE_VRS_***	***SE***	***TE_CRS_***	***TE_VRS_***	***SE***	***TE_CRS_***	***TE_VRS_***	***SE***
Mean	0.839	0.897	0.935	0.836	0.911	0.918	0.857	0.914	0.938	0.905	0.938	0.965	0.927	0.951	0.975
Median	0.936	0.926	0.958	0.909	0.945	0.988	0.925	1	0.943	1	0.999	0.969	0.914	1	0.961
Maximum	1	1	1	1	1	1	1	1	1	1	1	1	1	1	1
Minimum	0.594	0.652	0.742	0.545	0.629	0.781	0.60	0.616	0.879	0.679	0.744	0.817	0.682	0.764	0.772
SD	0.120	0.103	0.061	0.123	0.11	0.047	0.125	0.117	0.04	0.098	0.085	0.049	0.1	0.061	0.074
**Hospital ranking –n(%)**															
100%	18 (42.9)	20 (47.6)	21(50)	16 (38.1)	21 (50)	16 (38.1)	16 (38.1)	25 (59.5)	15 (35.7)	18 (42.9)	27 (64.3)	22 (52.4)	21 (50)	28 (66.7)	18 (42.9)
80–99.9%	14 (33.3)	15 (35.7)	20 (47.6)	15 (35.7)	13 (31)	25 (59.5)	15 (35.7)	9 (21.5)	27 (64.3)	15 (35.7)	10 (23.8)	20 (47.6)	15 (35.7)	13 (31)	21 (50)
60–79.9%	9 (21.4)	7 (16.7)	1 (2.4)	9 (21.4)	8 (19)	1 (2.4)	10 (23.8)	8 (19)	0 (0.0)	9 (21.4)	5 (11.9)	0 (0)	6 (11.3)	1 (2.4)	3 (7.1)
40–59.9%	1 (2.4)	0 (0.0)	0 (0.0)	2 (4.8)	0 (0.0)	0 (0.0)	1 (2.4)	0 (0.0)	0 (0.0)	0 (0.0)	0 (0.0)	0 (0.0)	0 (0.0)	0 (0.0)	0 (0.0)

CRS, constant return to scale; DEA, data envelopment analysis; SE, scale efficiency=TECRS/TEVRS; TECRS, overall technical efficiency from CRS DEA; TEVRS, pure technical efficiency from VRS DEA; VRS, variable return to scale.

### Overall TE (TE_CRS_)

For the years 2012, 2013, 2014, 2015 and 2016, out of the 42 hospitals, 18 (42.9%), 16 (38.1%), 16 (38.1%), 18 (42.9%) and 21 (50%) hospitals, respectively, were defined as technically efficient. Average TE_CRS_ was 0.839, 0.836, 0.857, 0.905 and 0.927, respectively. As shown in Table 3, over the period of the study (during before and after the implementation of HSEP), the TE_CRS_ increased from 0.839% in 2012 to 927% in 2016.

**Table 3:** Result from tobit regression analysis (N=42, year=2016)

***Variables***	***Coeffecient***	***SE***	***t***	***P***
Bed group				
Size 2 (150–250)	0.012	0.002	0.061	0.951
Size 3 (> 250)	0.016	0.006	0.076	0.989
Teaching status	0.047	0.040	1.16	0.253
Average Length of Stay	−0.113	0.010	−2.83	0.008
Bed Occupancy Rate	0.250	0.032	2.247	<0.001
Ratio of Nurses to Physicians	0.028	0.015	1.92	0.063
Ratio of Beds to Nurses	0.111	0.049	2.27	0.029
Constant	0.681	0.138	4.93	<0.001
Sigma	0.0842	0.0918		
Observations summary	0 left-censored observations			
	42 uncensored observations			
	0 right-censored observations			
Number of observations	42 sample hospitals in 2015			
Log likelihood	44.33			
ᵡ^2^	13.44			
Probability>ᵡ^2^	0.0975			

### Pure TE (TE_VRS_)

In 2012, 2012, 2014, 2015 and 2016, 22 (52.4%), 21 (50%), 17 (40.5%), 15 (35.7%) and 14 (33.3%) hospitals, respectively, operated inefficiently. Average TE_VRS_ was 0.897, 0.911, 0.914, 0.938 and 0.951, respectively, implying that if they run efficiently, the hospitals should decrease 10.3%, 8.6%, 7.6%, 6.2% and 4.9% of inputs for the same volume of outputs.

### Scale efficiency

As observed in Table 3, for the years 2012, 2013, 2014, 2015 and 2016, average SE was 0.935, 0.918, 0.938, 0.965 and 0.975, respectively. Nineteen (45.24%), 16 (38.09%), 15 (35.71%), 22 (52.38%) and 18 (42.86%) hospitals manifested CRS, indicating that they operated at their most productive size. Sixteen (30.09%), 19 (45.24%), 15 (35.71%), 22 (52.38%) and 18 (42.86%) showed IRS, suggesting that they should expand their scale to become scale efficient. Seven (16.66%), 7 (16.66%), 9 (21.43%), 6 (14.29%) and 1(2.38%) hospitals experienced DRS, meaning that they should scale down to become scale efficient.

### Results of tobit regression analysis

The results of Tobit regression analysis are shown in table 3. Factors such as teaching status (p=0.253), hospital size (*P*>0.05), respectively, were statistically insignificant with technical efficiency. ALOS has a negative impact on technical efficiency and were statistically significant (*P*=0.008). If the ALOS decreases by one day, hospitals’ expected efficiency score would increase by 0.113. Other variables, such as BOR, RONTP, and ROBTN, assumed positive signs with technical efficiency and were statistically significant (*P*<0.05). A unit increase in the BOR, RONTP and ROBTN would lead to an increase in hospital expected efficiency score by 0.250, 0.281 and 0.111, respectively.

## Discussion

Our study shows that approximately above 50% of hospitals experienced technical inefficient during 2012–2016. Therefore, there is need to improve their efficiency. In addition, efficient hospitals increase from 43% to 50% during 2012–2016. The average scale efficiency in hospitals was high and increased from 0.935 in 2012 to 0.975 in 2016. As a result, the low level of pure technical efficiency improved during the study period (from 0.897 to 0.951). The efficiency of studied hospitals was lower than those reported in other provinces of Iran. For example, the average of TE, pure TE and SE, in public hospitals in Tabriz during 2007–2010 was 0.984, 0.984 and 0.957 respectively (22). The existing difference can be caused by not the same selection of input and output variables and external environment.

BOR and ALOS were increased after the implementation of HSEP. This increase could be due to decrease in OOP payments, service quality promotion, and improvement of hoteling and accommodation services (14, 15). In Turkey, after the health care reforms, despite 18% increase in the number of beds between 2001 and 2006, the BOR and ALOS were fixed at 55% and 4.8 day, respectively (23). Following reforms in China, ALOS in teaching hospitals decreased from 12.1 in 2006 to 9.1 in 2010 (24). While, in another study in China, ALOS and BOR were increased (17).

Improving the technical efficiency of hospitals in the study is related to the increase in outputs in the HSEP, which is due to reduction in the share paid by patients and physicians’ more willingness to hospitalize patients due to tariff increase. It is recommended that payment policies be tailored between public and private sectors. Moreover, the status of distribution of resources between Tehran, as the Iranian capital, and other provinces is not so desirable (25, 26) and there is a need for revision and applying redistributive policies.

This study showed ALOS has had a negative impact on technical efficiency while BOR, RONTP and ROBTN had a positive impact. The ALOS in hospitals was 6.27 days that was different from other countries. In America, the ALOS was 3.5 days (27) and in China, it was 7.9 days (17). In China and Eritrea it is showed that longer ALOS leads to inefficiency of hospitals (17, 18). One reason for increasing ALOS in hospitals is teaching nature of their activity areas and majority of them being public (28). Therefore, managers must take some measures to reduce the ALOS, such as changes in payment policies, set clinical guidelines, creating competition, expanding the use of outpatient surgical procedures, innovations in medical technology and so on.

Positive impact of BOR on technical efficiency was in line with a study in China (17). BOR increased from 73% to 79%, which was higher than the standard set in Iran (BOR >70%) (9), but it was lower than the other countries. For example, BOR in China was 92% (17). In addition, they need to optimize the service delivery process as well. Reduction in the ALOS and increase in the number of patients after the implementation of HSEP have increased BOR. In Iran, after the implementation of HSEP, BOR had increased from 65.4% to 76.97% (9). The average of ROBTN was 2.12, while the value of this index for China was 2.59, and in Western countries, it was about 0.33 (29). The most important reason for this difference is unbalanced allocation of resources because most of these resources are concentrated in Tehran as the capital of Iran. This reason is also mentioned in China (17).

### Limitations

Our study had several limitations. First, this study was conducted only among public hospitals in Tehran, so the results cannot be generalized to all public hospitals in the country. Second, like many previous studies, case mixed index was not considered in the calculations of technical efficiency. Patients’ views were not included in the selection of outputs. In Iran, due to lack of a comprehensive database, assessing and collecting appropriate data associated with health outcomes, patient safety, mortality, quality of care and satisfaction as outputs is difficult. To improve the process of collecting the data, a database be created to address this limitation (30). In addition, due to limitation of DEA approach, technical efficiency scores are not moderated. A bootstrap DEA can be performed in future research to provide more exact findings.

## Conclusion

Technical efficiency of public hospitals has improved following the implementation of HSEP, yet there is capacity to improve the technical efficiency of hospitals. Increase in technical efficiency of sample hospitals has been due to increased output following the HSEP. Due to changes in tariffs and reduction of patients pay, the reason for this can be due to induction of demand from physicians. The sameness of provider and supervisor in Iran healthcare system strengthens the likelihood of proving this hypothesis. Accordingly, more studies in the field of quality and necessity of services provided seem essential. The ALOS is negatively associated with technical efficiency, whereas RONTP and ROBTN, and BOR have a significant positive impact on technical efficiency. Thus, we suggest that hospitals in Tehran can increase their technical efficiency by increasing BOR through shortening the average length of stay, proportioning the number of doctors, nurses, and beds along with service quality assurance.

## Ethical considerations

Ethical issues (Including plagiarism, informed consent, misconduct, data fabrication and/or falsification, double publication and/or submission, redundancy, etc.) have been completely observed by the authors.
